# The Role of Unfolded Protein Response in Human Intervertebral Disc Degeneration: Perk and IRE1-*α* as Two Potential Therapeutic Targets

**DOI:** 10.1155/2021/6492879

**Published:** 2021-03-24

**Authors:** Tianyong Wen, Peng Xue, Jinwei Ying, Shi Cheng, Yue Liu, Dike Ruan

**Affiliations:** ^1^Department of Orthopedic Surgery, Sixth Medical Center, Chinese PLA General Hospital, Beijing, China; ^2^Institute of Stomatology, First Medical Center, Chinese PLA General Hospital, Beijing, China

## Abstract

Inflammation plays a key role in intervertebral disc degeneration (IDD). The association between inflammation and endoplasmic reticulum (ER) stress has been observed in many diseases. However, whether ER stress plays an important role in IDD remains unclear. Therefore, this study is aimed at investigating the expression of ER stress in IDD and at exploring the underlying mechanisms of IDD, ER stress, and inflammation. The expression of ER stress was activated in nucleus pulposus cells from patients who had IDD (D-NPCs) compared with patients without IDD (N-NPCs); and both the proliferation and synthesis capacity were decreased by inducer tunicamycin (Tm) and proinflammatory cytokines. Pretreatment of NPCs with 4-phenyl butyric acid (4-PBA) prevented the inflammatory cytokine-induced upregulation of unfolded protein response- (UPR-) related proteins and recovered cell synthetic ability. Furthermore, proinflammatory cytokine treatment significantly upregulated the expression of inositol-requiring protein 1 (IRE1-*α*) and protein kinase RNA-like ER kinase (PERK), but not activating transcription factor 6 (ATF6). Finally, knockdown of IRE1-*α* and PERK also restored the biological activity of NPCs. Our findings identified that IRE1-*α* and PERK might be the potential targets for IDD treatment, which may help illustrate the underlying mechanism of ER stress in IDD.

## 1. Introduction

Low back pain (LBP) is a common disease, which may result in patients' disability and cause great economic burden to society [1]. Intervertebral disc degeneration (IDD) has been verified as a key factor for LBP [2]. Intervertebral disc (IVD) maintains homeostasis in physiological condition and degeneration can be triggered by several risk factors, such as abnormal mechanical stress, nutrient deficient, and aberrant inflammatory condition [3].

More importantly, inflammation is also considered an important contribution to IDD [4]. Inflammatory cytokines are significantly associated with the progression of IDD [5–7]. Furthermore, nucleus pulposus cells (NPCs) play an important role in maintaining nucleus pulposus (NP) tissue homeostasis, thereby facilitating the maintenance of spine biomechanics [8]. Therefore, the inflammatory microenvironment of IDD may have influence on the biological behaviors of NPCs.

The endoplasmic reticulum (ER) is responsible for folding and maturation of secreted and membrane proteins. Accumulation misfolded or unfolded proteins can compromise ER stress [9]. Restoring the normal function of ER can be achieved by the unfolded protein response (UPR), which involves three pathways—protein kinase RNA-like ER kinase (PERK), inositol-requiring protein 1 (IRE1-*α*), and activating transcription factor 6 (ATF6) [10]. Our previous studies demonstrated that chronic inflammation could lead to impairment of ER function, prolongation of ER stress, and defective osteogenic differentiation of PDLSCs [11]. However, there are rare studies reporting the role of ER stress and UPR in biological activity of human NPCs under inflammatory conditions in IDD.

In this study, we used two prominent proinflammatory cytokines (IL-1*β* and TNF-*α*) involved in IDD, aiming to mimic the inflammatory microenvironment. We isolated human nondegenerated NPCs (N-NPCs) and degenerated NPCs (D-NPCs) and observed the ER stress responses from both morphology and molecular biology level. We also tested the effects of IL-1*β* and TNF-*α* treatment on ER stress responses as well as biological activity potential.

## 2. Materials and Methods

### 2.1. Specimen Selection

Human NP tissues were obtained from 9 patients who underwent posterior discectomy for lumbar degenerative disease or other lumbar diseases (spine fracture, scoliosis, etc.). The details of all samples and patients are shown in Table 1. The NP tissues were sterilely stored in PBS solution. All surgical treatments were performed at the sixth medical center of PLA General Hospital (Navy General Hospital) from January 2016 to June 2017.

MRI was performed in all patients before surgery, and the degree of disc degeneration was assessed according to Pfirrmann's grading system based on T2-weighted MRI images [12]. Degenerated NPCs (D-NPCs) were isolated from the intervertebral disc classified as grade IV-V, while nondegenerated NPCs (N-NPCs) were isolated from the intervertebral disc classified as grade I-II.

The present study was approved by the medical ethics committee of PLA General Hospital (Navy General Hospital). Specific informed consent was obtained in all cases.

### 2.2. Cell Isolation and Culture

NP cells were isolated as described by Chelberg et al. [13]. Briefly, NP tissues were digested with 0.1% type II collagenase (Sigma-Aldrich, USA) in a serum-free medium overnight at 37°C with 5% CO_2_. The cells were cultured in a culture medium composed of DMEM/F12 (HyClone, USA) and 10% FBS for 7-10 days until confluent. To preserve the NPCs phenotype, primary cells were used when comparing expression levels in N-NPCs and D-NPCs, and the 1st and 2nd passages for other experiments.

ER stress response was triggered by 500 ng/ml tunicamycin (Tm) in DMEM containing 10% FBS for 12 hours. Our previous study showed that tunicamycin is more effective than thapsigargin (Peng Xue et al., Cell Death and Differentiation. 2016); thus, we chose TM as an ERs inducer. We tested the basal activation of the ER stress response by detection the main sensors (GRP78, CHOP, IRE-1*α*, PERK, and ATF6) on mRNA and protein levels.

A concentration of 5 ng/ml TNF-*α* and 1 ng/ml IL-1*β* was determined to efficiently simulate inflammatory microenvironment, and N-NPCs were treated with IL-1*β* and TNF-*α* in DMEM containing 10% FBS for 12 hrs. In another group, N-NPCs were pretreated by inhibitor 4-PBA (5 mmol/l) for 24 hrs, then treated with IL-1*β* and TNF-*α* as above. All Tm, 4-PBA, IL-1*β*, and TNF-*α* were purchased from Sigma-Aldrich, USA.

### 2.3. Transfection with Small Interfering RNA (siRNA)

To inhibit target genes expression, cells were transfected with PERK, IRE1, and ATF6 siRNA (Cell Signaling Technology, USA) conjugated with Lipofectamine 3000 (Invitrogen, USA) for 6 hrs. The cells were incubated at 37°C in an incubator for 24-48 h before further assay.

### 2.4. Transmission Electron Microscope (TEM)

After fixing in 4% glutaraldehyde and 4% paraformaldehyde (Sigma, both fix at Transives were diluted in phosphate buffered saline, pH 7.2) for one night, the NP tissue was postfixed in 2% osmium tetroxide and block-stained with 2% uranyl acetate, then dehydrated in a graded ethanol series, and embedded in situ in a LX-812 resin (Ladd Research Industries Inc., USA). Ultrathin sections were observed by a FEI Tecnai G12 Spirit BioTwin transmission electron microscope (FEI Company, USA) with an accelerating voltage of 100 kV. The digital images were captured by a CCD camera (Olympus-SIS, Germany).

### 2.5. Immunohistochemical (IHC) Analysis

The fragment of NP tissue was fixed in paraformaldehyde (4%), and paraffin sections (4 *μ*m thick) were prepared. IHC was performed using the Histostain Plus kit (Origin Technologies, China) with the anti-PERK, IRE1-*α*, and ATF6 antibody (Abcam, 1 : 500) at 4°C overnight. The sections were treated with 1% hyaluronidase for 60 min at 37°C (IRE1-*α* only), 0.25 units/ml protease-free chondroitinase ABC in 0.1 mol/l Tris acetate, 0.3% Triton for 30 min, and 3% H_2_O_2_ for 15 min at room temperature. The primary antibodies were diluted in goat serum and incubated overnight at 4°C. The primary antibodies were all rabbit anti-human against IRE1-*α* (monoclonal IgG 1/50; Cell Signaling Technology), PERK (monoclonal IgG 1/100; Cell Signaling Technology), and ATF6 (polyclonal IgG 1/100; Abcam). Detection was conducted using a DAB Horseradish Peroxidase Color Development kit (Origin Technologies, China).

### 2.6. Cell Proliferation

Cell proliferation of the 1st passage (P1) of N-NPCs, cells treated by TM, or inflammatory cytokines were evaluated using CCK-8 (Dojindo Laboratories, Japan). Briefly, 10 *μ*l of CCK-8 solution was added to each well of a 96-well plate containing 5000 cells. After incubating the plate at 37°C for 4 hrs, absorbance at 450 nm was measured using a microplate absorbance reader (Bio-Rad, USA). Cell proliferation was tested on days 1 to 7.

### 2.7. Quantification of mRNA and RT-PCR

The total RNA of every sample was extracted using the RNeasy mini kit (Qiagen GmbH, Germany) and converted to cDNA using Prime Script RT Master Mix (Takara Bio, Japan). RT-PCR was performed on real-time PCR (ABI PRISM 7000, USA), using the SYBR Green Master Mix reagent (Applied Biosystems, USA). The specific primers used for ER stress genes are shown in Table 2. GAPDH expression was used to normalize the expression of all genes. The relative expression levels of each gene were determined using the 2^-*ΔΔ*Ct^ method.

### 2.8. Western Blotting

Total protein of every sample was extracted using the radioimmunoprecipitation assay lysis buffer (Beyotime Institute of Biotechnology, China). Each sample was subjected to SDS-PAGE on a 10% gel and transferred to PVDF membranes. The membranes were blocked with 5% nonfat dry milk for 1 h at room temperature and then incubated with anti-*β*-actin (1/2000; Abcam), anti-grp78 (1/1000; Abcam), anti-PERK (1/1000; Cell Signaling Technology), anti-ATF6 (1 : 1000), anti-IRE1-*α* (1/1000; Cell Signaling Technology), anti-CHOP (1/1000; Abcam), anti-ColII (1/1000; Abcam), anti-Agg (1/1000; Abcam), and anti-Sox9 (1/1000; Abcam), at 4°C overnight. Then the membranes were incubated with the appropriate HRP-conjugated secondary antibody (1/2000) for 1 hr at room temperature, and signals were visualized using an enhanced chemiluminescence kit. Bands were detected and assessed through densitometric analysis.

### 2.9. Statistical Analysis

All quantitative data were presented as means ± standard deviation. The histological and western blot data are described qualitatively and shown as images. The values between the two groups were calculated by independent two-tailed unpaired Student's *t*-test, and the values between multiple comparisons were assessed with analysis of variance (ANOVA) with the Bonferroni correction. All statistical analyses were performed using Graph Pad Prism 5.0 (GraphPad Software, USA), and *P*-values less than 0.05 were considered statistically significant.

## 3. Results

### 3.1. Activation of ER Stress and UPR Target Genes in D-NPCs Isolated from Degenerative Microenvironment

**Figure 1:**
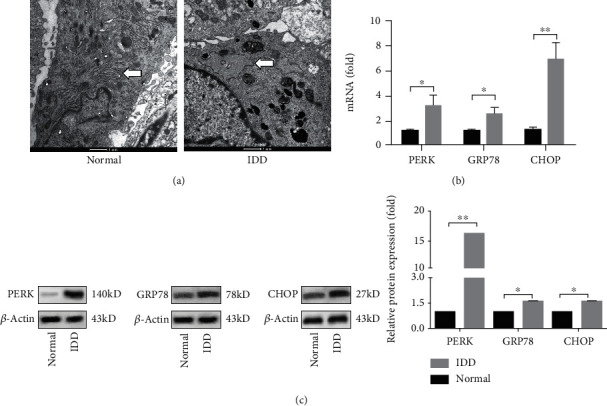
Activation of the UPR target genes in degenerated NPCs. (a) Transmission electron microscopy (TEM) images of endoplasmic reticulum (ER) in N-NPCs and D-NPCs. The morphology of ERs was showed with white arrows. The results showed more dilated and abundant ERs in D-NPCs. (b) The expressions of the UPR target genes (PERK, GRP78, and CHOP) in N-NPCs and D-NPCs were determined by real-time PCR. The results were normalized to GAPDH mRNA expression. (c) Western blot analysis showed the protein levels of PERK, GRP78, and CHOP in N-NPCs and D-NPCs. *β*-Actin was used as an internal control. The representative results were from three independent experiments. The error bars represent the SD from the mean values. ∗*P* < 0.05, ∗∗*P* < 0.01.

We isolated D-NPCs and N-NPCs. To investigate whether ER stress was induced in D-NPCs, we used TEM analysis to observe the endoplasmic reticulum. Our results showed that more dilated and abundant ER were observed in D-NPCs (Figure 1(a)). Next, validation by RT-PCR was performed for the representatives of the UPR target genes *PERK* (also called *EIF2AK3*), *GRP78* (*HSPA5/B*), and *CHOP* (*DDIT3*). The results of PCR indicated that the expression of UPR target genes was increased in D-NPCs compared to N-NPCs. Furthermore, we looked at whether the expression of UPR target genes was reflected in protein production by western blotting assay. The results shown in Figure 1(c) indicate that the UPR target genes expressions were upregulated in D-NPCs, indicating that degeneration may induce ER stress and subsequently produce UPR in NPCs.

### 3.2. UPR Activation Leads to the Decreased Biological Activity of NPCs

**Figure 2:**
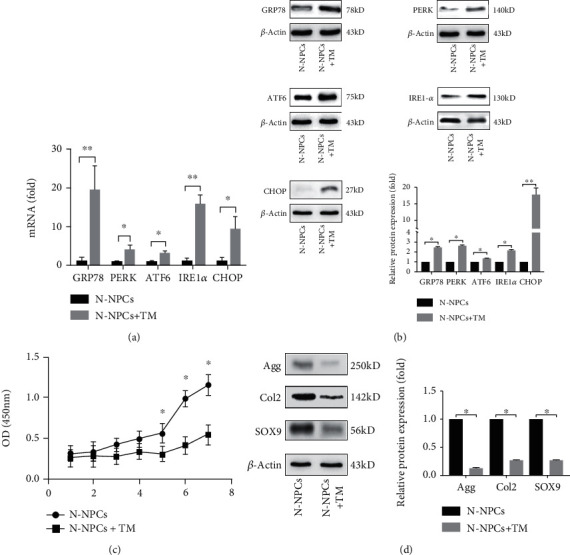
UPR activation leads to the decreased biological activity of NPCs. The expression of UPR genes in NPCs treated by Tm was analyzed by real-time PCR (a) and western blot (b). (c) Cell proliferation was evaluated by the CCK-8 assay at days 1 to 7 after TM treatment. (d) The expression of Agg, Col2, and SOX9 following the exposure of NPCs to Tm was confirmed by western blot. *β*-Actin was used as an internal control. The representative results were from three independent experiments. The error bars represent the SD from the mean values. ∗*P* < 0.05, ∗∗*P* < 0.01.

Since the chronic inflammation activates UPR response as well as impairs the biological activity of NPCs in IDD, we want to know whether activated UPR also leads to the impaired biological activity of NPCs. Then, we treated NPCs with UPR inducer, tunicamycin (Tm) for 12 hrs. The greatest increase of expression was observed in *GRP78*, *IRE1-α*, and *CHOP*, and increased expression was also noted with *PERK* and *ATF6* (Figure 2(a)). Western blot also indicated that the three main branches of UPR, PREK, IRE1-*α*, and ATF6 expression significantly increased, as well as GRP78 and CHOP (Figure 2(b)). As to proliferation capacity assay, similar growth tendencies were observed in both groups. According to the OD values, a continuous increase was observed from days 1 to 7, and a little decrease was formed at day 5 in the TM group. However, a significant higher proliferation capacity was found in the control group at the last 3 time points (Figure 2(c)).

Our previous study showed defective biological activity of D-NPCs [14]. To know whether activated ER stress results in synthetic impairment of NPCs, we tested the biological activity of N-NPCs treated by Tm for 12 hrs. Increased UPR may contribute to a decrease in Aggrecan (Agg), Collagen II (Col2), and Sox9 after 24 hrs of induction (Figure 2(d)). These results indicate that strong respondence to acute ER stress could be observed in NPCs, which could have an influence on biological activity.

### 3.3. Inflammation Triggers UPR and Impairs Biological Activity of NPCs

**Figure 3:**
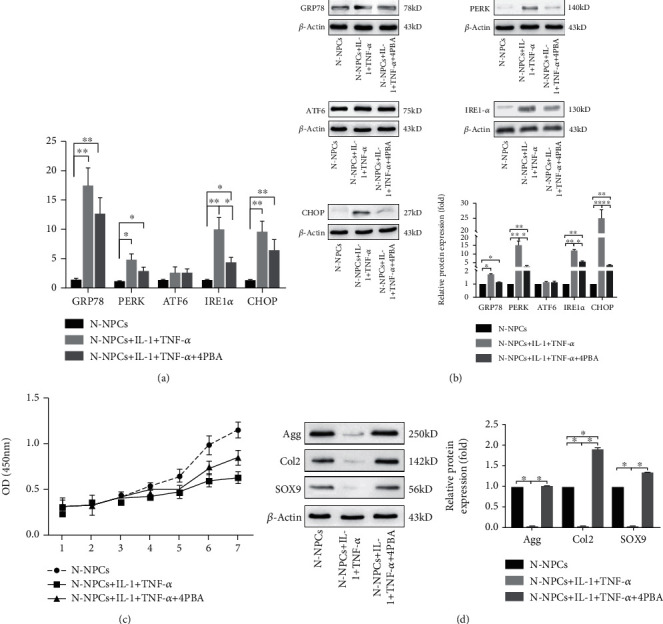
Inflammation triggers UPR and impairs biological activity of NPCs. The expression of UPR gene in NPCs treated by IL-1 and TNF-*α* synergistically without/with 4-PBA was analyzed by real-time PCR (a) and western blot (b). (c) Cell proliferation was evaluated by the CCK-8 assay at days 1 to 7 after IL-1 and TNF-*α* synergistically without/with 4-PBA treatment. (d) The expression of Agg, Col2, and SOX9 following the exposure of NPCs to IL-1 and TNF-*α* synergistically without/with 4-PBA was confirmed by western blot analysis. *β*-Actin was used as an internal control. The representative results were from three independent experiments. The error bars represent the SD from the mean values. ∗*P* < 0.05, ∗∗*P* < 0.01.

To know whether inflammatory stimulation induces UPR in NPCs, we used IL-1*β* and TNF-*α*, which were found highly expressed in NPCs in IDD patients [14], to treat N-NPCs and observe the UPR in the cells. However, after the 12 hrs stimulation of IL-1*β* and TNF-*α* synergistically, our results showed that the expressions of *GRP78*, *PERK*, *IRE1-α*, and *CHOP* were increased. Then, we pretreated NPCs with an UPR inhibitor 4-PBA for 24 hrs, while *GRP78*, *PERK*, *IRE1-α*, and *CHOP* of D-NPCs were decreased after the 4-PBA treatment, but the expression of *ATF6* was similar in all the three groups (Figure 3(a)). The western blot had similar results, indicating that PERK and IRE1-*α* expression in the inflammatory response are upregulated in D-NPCs; however, the ATF6 expression was similar (Figure 3(b)).

Regarding the proliferation capacity, similar growth tendencies were observed in all the three groups. A continuous increase was observed from days 1 to 7, which was similar with the CCK8 results of the TM-treated group (Figure 2(c)). However, a slightly higher proliferation capacity was found in IL-1*β*, TNF-*α*, and 4-PBA groups at the last 2 time points (Figure 3(c)).

Next, IL-1*β* and TNF-*α* were used to treat NPCs synergistically in biological activity. Western blot showed that Agg, Col2, and Sox9 were decreased. However, the biological activity of N-NPCs was rescued after 4-PBA treatment, since the synthetic marker proteins were recovered (Figure 3(d)).

Taken together, these results indicate that the ER stress response is not globally or fully activated in the inflammatory environment of NPCs.

### 3.4. UPR Activation Regulates Biological Activity of NPCs through PERK and IRE1-*α* Pathways

**Figure 4:**
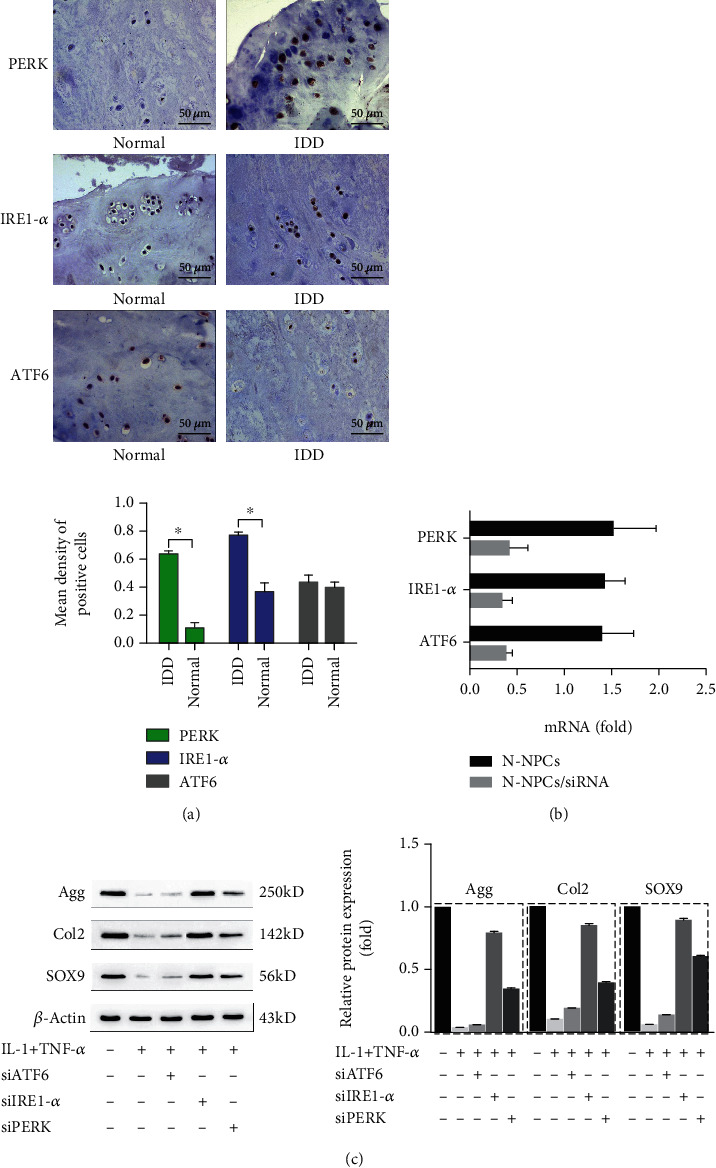
UPR activation regulated the synthesis of NPCs through the PERK and IRE1-*α* pathways. (a) The expression of PERK, ATF6, and IRE1-*α* proteins in degenerated and nondegenerated NP tissues was detected by IHC. Representative images are presented at the indicated magnifications. Scale bar, 50 *μ*m. Compared with the controlled group, the presence of PERK and IRE1-*α* was significantly increased in degenerated tissues, whereas ATF6 was similar. (b) The expression of ATF6, IRE1-*α*, and PERK was downregulated after being transfected with PREK, ATF6, and IRE1-*α* siRNA as assayed by real-time PCR. (c) The expression of Agg, Col2, and SOX9 was significantly increased after knockdown of PERK and IRE1-*α* than ATF6 as assessed by western blot. *β*-Actin was used as an internal control. The representative results were from three independent experiments. The error bars represent the SD from the mean values.

The basal activation status of the three main branches of UPR was monitored in D-NPCs. The presence of PERK, IRE1-*α*, and ATF6 was determined by immunohistochemical (IHC) in nondegenerated and degenerated NP tissues. The presence of PERK and IRE1-*α* was determined by IHC in D-NPCs, whereas ATF6 was similar in both N-NPCs and D-NPCs (Figure 4(a)).

In order to know whether impaired biological activity of NPCs is affected by PERK and IRE1-*α* pathway in chronic inflammatory microenvironment, we used siRNAs to knockdown three sensors of ER stress, respectively, and observed the biological activity of N-NPCs. The results showed decreased expressions of PERK, IRE1-*α*, and ATF6 by real-time PCR (Figure 4(b)). We found that the synthesis proteins of Agg, Col2, and Sox9 were significantly increased in PERK and IRE1-*α* siRNA-transfected D-NPCs, respectively, compared with the controlled group caused by IL-1*β* and TNF-*α* treatment. However, the biological activity of NPCs was not rescued after the transfection with ATF6 siRNA (Figure 4(c)).

As described above, we demonstrate that ER stress regulates biological activity of NPCs through PERK and IRE1-*α* pathway.

## 4. Discussion

In this study, we uncovered a previously unrecognized link between the UPR pathway and IDD. For the first time, we report that inflammatory factors activate the UPR sensors (PERK, IRE1-*α*) and then impair the biological activity of NP cells, as summarized in the Graphical Abstract. According to our data, when the two pathways were blocked, the cell function can be recovered. The results indicated PERK and IRE1-*α* may be the important mediators of UPR and IDD.

IDD is a degenerative status of spine with aging, which mainly starts from nucleus pulposus. Loss of NPCs and decrease of protein synthesis could result in the degradation of ECM and break the homeostasis in IVD [15], which would cause discogenic low back pain, disc herniation, etc. Despite the increasing prevalence of IDD-related diseases and consequently high economic burden, both conservative and surgical treatments mainly focus on the relief of pain and reconstruction of stability, not on the etiology of degeneration. Current studies explore new strategies for restoring the function and homeostasis of degenerated discs via inhibition of inflammation, prevention of premature aging, and improvement of the ECM content [2].

Increasing attention has been paid on the research of inflammatory reactions in NPCs, though several factors have been found to play a key role in degeneration of NPCs [16, 17]. ER stress is an identified subcellular pathological process, which has been found participating in many chronic inflammatory diseases (such as rheumatoid arthritis and diabetes) [18, 19]. The UPR is a following response to ER stress, serving to compromise the stress that results from the presence of misfolded proteins in an attempt to restore homeostasis. It is activated via three pathways: PERK, IRE1-*α*, and ATF6 [20]. Therefore, we speculated that there may be a relationship between IDD and UPR. Similar with IDD which begins with cell degeneration, osteoarthritis (OA) is a progressive disease of the joints resulting in the degeneration of articular cartilage. Some studies showed the expression of some UPR genes had been investigated in OA chondrocytes. A study showed that chondrocytes from human OA cartilage displayed ER stress and that ER stress and apoptosis were increased during the progression of OA. The authors found XBP1 mRNA splicing, which represented a protective ER stress response, increased in moderate OA cartilage, but not in mild or severe cartilage [21]. However, one research demonstrated that the level of ER stress is not significantly increased in OA chondrocytes [22]. But in their results, downregulating PERK expression increased COL1a1 and suppressed COL2a1 expression which impacted ECM of cartilage. Another study reported the intracellular accumulation of AGE-induced chondrocytes apoptosis via ER stress [23]. However, few investigations have identified the relationship between ER stress and the degeneration of NPCs. A study reported disc cell apoptosis mediated simultaneously by ER and mitochondria played a potent role in IDD, but no more further molecular results about UPR were presented [24]. In our present research, we found the level of ER stress in D-NPCs is obviously higher than N-NPCs. The morphology of ER also showed more dilated and abundant in D-NPCs by TEM. Then, we activated the ER stress and found the proliferation and synthesis of NPCs all declined. These findings indicate the ER stress may participate in the process of IDD.

IL-1*β* and TNF-*α* are the classical primary proinflammatory cytokines of IDD; and their progression plays a role in the inhibition of anabolic process and proliferation required for IVD maintenance [16, 25, 26]. In this study, we combined IL-1*β* and TNF-*α* to stimulate the inflammatory status in vitro and also demonstrated that IL-1*β* and TNF-*α* inhibited the NPCs proliferation and synthesis. Meanwhile, the UPR was activated. When the ER stress inhibitor 4-PBA was added to the proinflammatory factor-treated cells, the biological activity partially recovered. These evidences further illustrated the inflammatory environment induced IDD via ER stress.

The results of RT-PCR and Western blot indicated that (1) the signaling pathways of UPR had been activated partially and (2) the mRNA and protein level of IRE1-*α* and PERK are significantly higher than the controlled group, which were consistent with the IHC in the IDD tissues from patients. All those findings indicated that IL-1*β* and TNF-*α* affected the biological activity of NPCs through the PERK and IRE1-*α* pathways. To confirm this opinion, we used the gene-silencing technique to block the UPR pathway separately and found the silence of PERK and IRE1-*α* caused the upregulation of synthesis activity, especially the silence of IRE1-*α*. These results accorded with another research of OA [22]. However, as reported in other diseases, PERK and IRE1-*α* played dual roles depending on whether they were activated by physiological factors or by ER stress, and the cellular responses were different depending on the stimulating factors [27, 28].

In conclusion, we found the ER stress was activated in degenerated IDD. The cell biological activity of NPCs can be partly preserved by the block of the two UPR pathways when treated by TNF-*α* and IL-1, which indicated inflammation may induce IDD by the PERK and IRE1-*α* pathways. Our study identifies the two pathways as the potential target for molecular therapeutics of IDD.

## Figures and Tables

**Table 1:** Details of samples and patients.

Case no.	Diagnosis	Disc level	Pfirrmann's grade	Gender	Age
1	Disc herniation	L4-5	II	M	33
2	Disc herniation	L4-5	IV	M	52
3	Disc herniation	L4-5	V	F	34
4	Disc herniation	L4-5, L5-S1	V	M	22
5	Vertebral fracture	L3-4	II	F	43
6	Disc herniation	L4-5	IV	M	17
7	Lumbar stenosis	L3-4	IV	M	50
8	Disc herniation	L4-5	II	M	28
9	Scoliosis	L1-2	I	F	21

**Table 2:** Sequences of primers used in real-time RT-PCR.

Gene	Primer sequences(5′-3′)
PERK	Forward: GTCCGGAACCAGACGATGAG Reverse: GGCTGGATGACACCAAGGAA
ATF6	Forward: CGGAGTATTTTGTCCGCCTG Reverse: GCTGCTTCCAATTGCAGCTC
IRE1-*α*	Forward: CCTAGTCAGTTCTGCGTCCG Reverse: TTCCATCCAGCGTTGACACA
GRP78	Forward: TCAAGTTCTTGCCGTTCAAGG Reverse: AAATAAGCCTCAGCGGTTTCTT
CHOP	Forward: CAAGAGGTCCTGTCTTCAGATGA Reverse: TCTGTTTCCGTTTCCTGGTTC

## Data Availability

All data generated or analyzed during this study are included in this article.
